# A Structurally Characterized Organometallic Plutonium(IV) Complex

**DOI:** 10.1002/anie.201701858

**Published:** 2017-03-30

**Authors:** Christos Apostolidis, Olaf Walter, Jochen Vogt, Phil Liebing, Laurent Maron, Frank T. Edelmann

**Affiliations:** ^1^ European Commission, Joint Research Centre, Directorate G—Nuclear Safety and Security P.O. Box 2340 76125 Karlsruhe Germany; ^2^ Chemisches Institut der Otto-von-Guericke-Universität Magdeburg Universitätsplatz 2 39106 Magdeburg Germany; ^3^ Laboratoire de Physique et Chimie des Nanoobjets (LPCNO) Université de Toulouse/INSA/CNRS (UMR5215) 135 avenue de Rangueil 31077 Toulouse cedex 4 France

**Keywords:** plutonium, sandwich complexes, silyl group migration, X-ray crystallography

## Abstract

The blood‐red plutonocene complex Pu(1,3‐COT′′)(1,4‐COT′′) (**4**; COT′′=η^8^‐bis(trimethylsilyl)cyclooctatetraenyl) has been synthesized by oxidation of the anionic sandwich complex Li[Pu(1,4‐COT′′)_2_] (**3**) with anhydrous cobalt(II) chloride. The first crystal structure determination of an organoplutonium(IV) complex revealed an asymmetric sandwich structure for **4** where one COT′′ ring is 1,3‐substituted while the other retains the original 1,4‐substitution pattern. The electronic structure of **4** has been elucidated by a computational study, revealing a probable cause for the unexpected silyl group migration.

The highly radioactive transuranium element plutonium (Pu) was first produced in 1940 by Glen Seaborg et al. by deuteron bombardment of ^238^U.^1^ Because of its numerous applications in nuclear technology, the inorganic chemistry of this fascinating actinide metal is well‐investigated. In sharp contrast, organometallic Pu complexes comprising direct Pu−C bonds are scarce because there are few laboratories in the world that are able to handle air‐sensitive compounds of radioactive and toxic Pu safely. Moss‐green tris(cyclopentadienyl)plutonium(III), PuCp_3_ (Cp=η^5^‐cyclopentadienyl), was the first well‐defined organoplutonium ever reported. It was synthesized by Fischer et al. from PuCl_3_ and BeCp_2_ in a melt at 70 °C.^2^ The synthesis of PuCp_3_ was later improved,^3^, ^4^ and the range of Pu cyclopentadienyl complexes was expanded to include a series of related Pu^IV^ derivatives.^5^, ^6^, ^7^, ^8^, ^9^ More recently, cyclopentadienyl complexes of Pu and other transuranium elements have mostly been the subject of theoretical studies.^10^, ^11^, ^12^, ^13^, ^14^, ^15^, ^16^, ^17^, ^18^, ^19^, ^20^

Following the discovery of iconic uranocene U(COT)_2_ in 1968,^21^ the first unsubstituted bis(cyclooctatetraenyl) complexes of transuranium metals, An(COT)_2_ (An=Np, Pu; COT=η^8^‐cyclooctatetraenyl), were reported in the early 1970s by Karraker and Streitwieser Jr. et al.^22^, ^23^ Plutonocene, Pu(COT)_2_, was prepared by addition of solid [NEt_4_]_2_[PuCl_6_] to a tetrahydrofuran (THF) solution of K_2_COT and isolated as an air‐sensitive solid giving cherry‐red solutions in toluene.^22^ Subsequently, anionic complexes of the type K[An(COT)_2_] (An=Np, Pu) were reported^24^, ^25^ and the series of neutral sandwich complexes was extended to a number of ring‐substituted derivatives such as An(EtCOT)_2_, An(^*n*^BuCOT)_2_, and An(Me_4_COT)_2_ (An=Pa, Np, Pu).^25^, ^26^, ^27^, ^28^ From the very beginning until today, the magnetic and electronic properties of these actinocenes and related sandwich complexes intrigued theoretical chemists.^29^, ^30^, ^31^, ^32^, ^33^, ^34^, ^35^, ^36^, ^37^, ^38^ For example, it was shown that, while 5f contributions to covalency in these actinocenes are smaller in magnitude than 6d contributions, the variation in covalency is almost entirely accounted for by the variation in the 5f contribution.^36^

Remarkably, while the chemistry of uranocene and thorocene (Th(COT)_2_) is still being investigated actively,^39^, ^40^, ^41^, ^42^ preparative investigations involving plutonocene and neptunocene and their derivatives have been more or less discontinued since the 1980s because of extensive radiation safety regulations.^39^ Meanwhile, following the pioneering work of Cloke et al.,^43^, ^44^ silylated COT ligands such as 1,4‐bis(trimethylsilyl)cyclooctatetraenyl (1,4‐COT′′) or 1,4‐bis(triisopropylsilyl)cyclooctatetraenyl) have become very popular in organolanthanide and organoactinide chemistry. In many cases, anionic and neutral sandwich complexes comprising these bulky COT ligands exhibit better solubility and higher crystallinity than their unsubstituted congeners.^45^, ^46^ There is only one early preliminary report in which the compound Np(COT′′′)_2_ (COT′′′=1,3,5‐tris(trimethylsilyl)cyclooctatetraenyl) was mentioned.^13^ This dark‐red neutral neptunium(IV) sandwich complex was obtained in high yield (88 %) by treatment of NpCl_4_ with 2 equiv of K_2_(COT′′′)⋅2 THF, but its characterization was limited to elemental analysis and IR data.^47^ Herein, we report an organometallic building block approach to the neutral plutonocene complex Pu(1,3‐COT′′)(1,4‐COT′′) (**4**), as well as a detailed structural and theoretical investigation of this new transuranium sandwich complex.

The method leading to compound **4** is outlined in Scheme 1. The synthetic strategy consists of a building‐block approach whereby **4** is built from the bottom up. The synthesis starts with 1,4‐bis(trimethylsilyl)cycloocta‐2,5,7‐triene (**1**), which is readily available according to Cloke et al., by treatment of cycloocta‐1,5‐diene with *n*‐butyllithium/*N*,*N*,*N*′,*N*′‐tetramethylethylenediamine followed by reaction of the intermediate Li_2_(COT′′) with chlorotrimethylsilane.^44^ Metalation of **1** with 2 equiv of *n*‐butyllithium in THF solution affords the intermediate **2**, which was treated with 0.45 equiv of anhydrous plutonium(III) chloride, resulting in formation of the green anionic sandwich complex Li[Pu(1,4‐COT′′)_2_] (**3**). The final step, that is, oxidation to the neutral Pu^IV^ sandwich, was inspired by our previously discovered method, which employed anhydrous CoCl_2_ as a mild oxidant to convert the anionic lanthanide(III) sandwich complexes [Ln(COT′′)_2_]^−^ into the linear neutral triple‐decker sandwich species Ln_2_(COT′′)_3_.^48^, ^49^, ^50^, ^51^ Thus, after complete removal of THF, the green‐brown residue was redissolved in toluene. Addition of 0.5 equiv of anhydrous CoCl_2_ caused an immediate color change to red, indicative of the formation of Pu^IV^ species. To date, organometallic complexes of Pu have been reported in the literature for the oxidation states Pu^III^ and Pu^IV^. The organometallic Pu^III^ complexes are generally described as green in color,^2^, ^24^ whereas the Pu^IV^ complexes are normally red to brown.^5^, ^6^, ^8^, ^22^, ^26^, ^27^ After reflux, insoluble material (Co and LiCl) was removed by filtration; the filtrate was evaporated to dryness and the red product extracted with *n*‐pentane. Removal of the *n*‐pentane left a dark‐red oil which solidified after standing at room temperature for 2 d to give low‐melting, blood‐red crystals of **4** in approximately 85 % isolated yield.

**Scheme 1:**
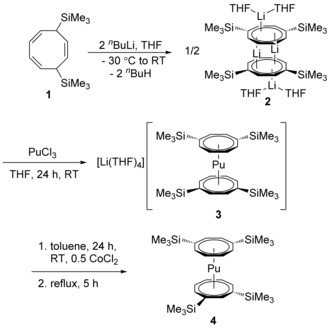
Synthetic route to the Pu^IV^ sandwich complex **4** starting from 1,4‐bis(trimethylsilyl)cycloocta‐2,5,7‐triene (**1**).

The Pu^IV^ sandwich complex **4** was characterized by NMR spectroscopy and single‐crystal X‐ray diffraction. The ^1^H NMR spectrum of **4** resembled that of the corresponding uranocene complex U(1,4‐COT′′)_2_, apart from a very different chemical shift range.^46^, ^52^ According to the results of the X‐ray structure analysis, a complicated NMR spectrum was expected resulting from two COT rings with different substitution patterns. However, this was not observed in practice. The following assignment has been confirmed by room temperature CH correlated spectroscopy (Supporting Information). One resonance is observed at 0.97 ppm (relative intensity 18) for the SiMe_3_ protons, indicating free rotation of the COT rings at room temperature and potentially rapid chemical exchange. Three multiplets (relative intensity 2 each) at 10.19, 10.08, and 9.48 ppm were assigned to the COT ring protons. In accordance with the X‐ray analysis, which shows that one of the COT rings is substituted at the 1,3‐positions, a very broad resonance observed at 9.78 ppm with a relative intensity is assigned to the proton in the 2‐position of this ring. Additional signals were not resolved. The low number of signals supports a potential chemical exchange. However, radiation safety issues arising from low‐ or high‐temperature NMR experiments could not be performed. Furthermore, the lifetime of the title complex is limited so that all NMR experiments had to be completed within 6 h.

An X‐ray diffraction study of **4** confirmed the presence of a neutral Pu^IV^ sandwich complex with the two COT′′ rings in a virtually coplanar arrangement (Figure 1). The angle between the Pu atom and the ring centroids is almost linear (176.7°), and is identical to that in the neutral cerocene sandwich Ce(COT′′′)_2_ complex (176.1°)^53^ or the corresponding U and Th complexes.^46^, ^52^ The Pu−C distances are in a range between 2.60(1) and 2.70(1) Å, with Pu−COT′′ (ring centroid) distances of 1.89 and 1.90 Å, respectively. These values are about 0.10 Å shorter than those for the corresponding Th complex and about 0.03–0.04 Å shorter than that for the U analogue. On the basis of our data it remains unclear whether this discrepancy is caused by ion radius shrinkage arising from actinide contraction and/or changes of covalency in the bonding.^46^ The most remarkable structural feature of **4**, however, is the 1,3‐substitution pattern in one of the coordinated COT′′ ligands. All known preparations of f‐element COT′′ sandwich and half‐sandwich complexes start with the precursor 1,4‐bis(trimethylsilyl)cycloocta‐2,5,7‐triene (**1**). It is generally assumed that the 1,4‐substitution pattern is retained upon formation of various forms of Li_2_(COT′′), as exemplified by the structurally characterized derivatives Li_4_(1,4‐COT′′)_2_(THF)_4_ (**2**)^54^ and Li_2_(DME)_2_(1,4‐COT′′).^55^

**Figure 1:**
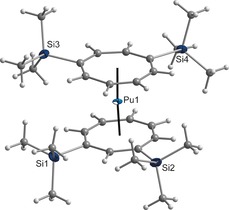
Molecular structure of compound **4** in the crystalline state. Displacement ellipsoids of Pu and Si drawn at the 50 % probability level. Selected bond lengths [Å]: Pu−C 2.60(1)–2.70(1), Pu−COT′′ (centroid) 1.89 and 1.90. Selected bond angle [°]: COT′′‐Pu‐COT′′ 176.7.

1,4‐Substitution was also found in all previously reported lanthanide half‐sandwich and sandwich complexes [(1,4‐COT′′)Ln(μ‐Cl)(THF)]_2_, [Li(THF)_4_][Ln(1,4‐COT′′)_2_], and [Li(DME)_3_][Ln(1,4‐COT′′)_2_], as well as in the triple‐decker sandwich complexes Ln_2_(1,4‐COT′′)_3_ (Ln=Nd, Dy, Er).^46^, ^48^, ^49^, ^50^, ^51^, ^52^, ^56^, ^57^, ^58^ However, there are a few remarkable reports in the literature that indicate the triorganosilyl groups in these bulky bis(silylated) COT ligands can migrate under certain conditions; thus far, this process is not fully understood. For example, the first case of silyl group migration to the 1,3‐positions was observed when the very bulky 1,4‐bis(triphenylsilyl)cyclooctatetraenyl dianion (COT^BIG^) was employed as a ligand in cerium sandwich complexes. Surprisingly, oxidation of the anionic cerium(III) sandwich [Li(DME)_2_][Ce(1,4‐COT^BIG^)_2_] with AgI resulted in formation of the neutral cerocene Ce(1,3‐COT^BIG^)_2_.^2^, ^3^, ^59^ On the other hand, the triple‐decker sandwich complexes Ho_2_(COT′′)_3_ was found to be the isomers (1,4‐COT′′)Ho[μ‐η^8^:η^8^‐1,5‐COT′′]Ho(1,4‐COT′′) in which rearrangement of the central COT′′ ligand to the 1,5‐regioisomer had taken place.^50^, ^60^

In addition to the X‐ray diffraction analysis, we also carried out a computational study on Pu(1,3‐COT′′)(1,4‐COT′′)_2_ (**4**), with the idea of understanding the cause of the silyl migration. Computations of Pu‐containing molecules are still rather scarce in the literature, mainly because of the number of electrons contained in such systems and the treatment of relativistic/correlation effects. Previous studies^33^, ^34^, ^35^, ^36^ have shown that the metal−carbon bonds in the highly symmetric plutonocene molecule have a multiconfigurational character with significant contribution of static as well as dynamic electron correlations. In view of the low point symmetry and the considerable dimension of the Pu(1,3‐COT′′)(1,4‐COT′′) molecule, compromises in the selection of basis sets and the chosen methods had to be made. Thus, second order Møller–Plesset perturbation theory (MP2), covering significant parts of dynamic electron correlation, in combination with restricted open shell Hartree–Fock (ROHF) single reference calculations, could be considered most appropriate for geometry optimizations of compound **4**. On the other hand, it was recently demonstrated by the Maron group that DFT can properly account for the geometry, bonding, and reactivity of complexes of transuranic metals such as Pu^61^, ^62^ or berkelium.^63^ Based on reported computational studies of Pu systems, different computational methods (namely ROHF, MP2, and DFT) were used. For **4**, the ground state was assumed to have the same multiplicity as nonsubstituted plutonocene with a ground state of ^5^A_g_.^14^, ^15^, ^34^, ^35^, ^61^

Structural data for the Pu(1,3‐COT′′)(1,4‐COT′′) molecule optimized at the ROHF and the MP2 level can be found in Table S9 (Supporting Information) alongside the experimental data. The optimum structure from the MP2 geometry optimization is shown in Figure 2. While the Pu−ring distances obtained from ROHF optimized geometry calculations exceed the experimental values by about 0.08 Å, the MP2 Pu−ring distances are 0.05 Å smaller than the measured values. In both cases the discrepancies might arise from the neglect of the multiconfigurational character of the metal−ligand bonds. The MP2 angle enclosed by the ring centroids and the Pu atom is 177.2° and therefore in reasonable accordance with the experiment.

**Figure 2:**
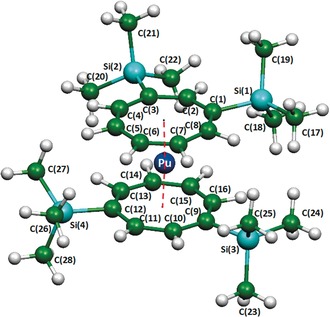
Structure of Pu(1,3‐COT′′)(1,4‐COT′′) (**4**) obtained from MP2/LANL2DZ(d,p) geometry optimizations. The black dots linked by red dashed lines passing through the central Pu atom mark the positions of the ring centroids.

This slight tilt displaces the rather bulky silyl groups from each other. Concerning the experimentally observed ring distortions: steric strain resulting from the presence of SiMe_3_ groups is compensated by a moderate torsion of the rings out of the eclipsed position by about 2.5°. The geometry optimization was carried out using a method described by us in conjunction with other actinide complexes.^63^, ^64^, ^65^, ^66^ The main geometrical parameters are in excellent agreement with those of Pu(COT)_2_ listed in Table S8 (Supporting Information), with a Pu−COT′′ distance of 1.87 Å, C−C bond lengths in the COT′′ moiety of 1.41 Å, and C−H bond lengths of 1.09 Å. As DFT properly reproduces the geometry, silyl migration was considered to find possible explanations for this rather uncommon phenomenon, keeping in mind that this does not occur in the U analogue. Therefore, geometry optimization was carried out at the DFT level on the exclusively 1,4‐substituted plutonocene complex (analogous to the uranocene complex); that is without the silyl migration on one COT ligand. Interestingly, this complex was also a minimum on the Potential Energy Surface (PES), but slightly less stable than the experimentally determined energy (3.0 kcal mol^−1^). Therefore, the migration is not due to greater steric effects on the Pu complex relative to those acting on the U complex. A possible explanation for silyl migration is put forward by the molecular orbital diagram of the two complexes. Indeed, as expected from other studies on organoactinide complexes, the highest occupied orbitals are two δ‐orbitals (not the singly occupied molecular orbital (SOMO) that are 5f orbitals), which describe the interaction between Pu and the COT′′ ligand (Figure 3).

**Figure 3:**
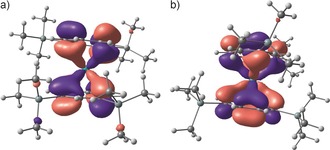
δ‐Bonding orbital for a) the geometry equivalent to the X‐ray structure of Pu(1,3‐COT′′)(1,4‐COT′′) (**4**) and b) the geometry equivalent of Pu(1,4‐COT′′)_2_ to the uranocene analogue.^52^

Scrutinizing the molecular orbitals, it appears that there is a contribution from the silyl group to the δ‐bond, which is in line with a hyperconjugation effect (Figure 3). Such a hyperconjugation is not observed in the case of the uranocene derivative U(COT′′)_2_,^43^, ^52^ where calculations indicate that the structure with exclusively 1,4‐bis(trimethylsilyl)‐substituted COT ligands is more stable than that with a mixed 1,4‐/1,3‐substitution pattern by 3.5 kcal mol^−1^ (Supporting Information, Figure S4a,b). This is a striking difference between U and Pu, which is explained by the molecular orbital diagrams of the two species (Supporting Information, Figure S6c,d). Indeed, because of the lower energy of the 5f atomic orbitals for Pu, the δ‐orbitals are higher in energy than the unpaired electrons, which is not the case for the U complex. Therefore, the highest energy of the δ‐orbitals in Pu allows contribution from the antibonding Si−C orbital of the silyl substituents (negative hyperconjugation). This stabilizing effect might be the cause for the silyl migration.

In summary, we succeeded in the synthesis of the neutral Pu^IV^ sandwich complex Pu(1,3‐COT′′)(1,4‐COT′′) (**4**). The synthetic protocol (that is, oxidation of the anionic Pu^III^ sandwich complex Li[Pu(1,4‐COT′′)_2_] (**3**) with anhydrous CoCl_2_) was inspired by our previously discovered method to convert the anionic lanthanide(III) sandwich complexes [Ln(COT′′)_2_]^−^ into the linear neutral triple‐decker sandwich species Ln_2_(COT′′)_3_. Blood‐red crystals of **4** were obtained in 84 % yield after extraction with *n*‐pentane. Compound **4** is the first organometallic Pu^IV^ complex structurally characterized by X‐ray diffraction.^67^ The crystal structure determination of **4** revealed a sandwich structure with one 1,3‐substituted COT′′ ring while the other ring retains the original 1,4‐substitution pattern. The electronic structure of **4** has been elucidated by computational methods, revealing a plausible cause for this unexpected silyl group migration. These results may contribute to a more profound knowledge of the fascinating properties of transuranium organometallic compounds.

## Conflict of interest

The authors declare no conflict of interest.

## Supporting information

As a service to our authors and readers, this journal provides supporting information supplied by the authors. Such materials are peer reviewed and may be re‐organized for online delivery, but are not copy‐edited or typeset. Technical support issues arising from supporting information (other than missing files) should be addressed to the authors.

Supplementary
