# Supplementary material for: A Structurally Characterized Organometallic Plutonium(IV) Complex
Source: Angew Chem Int Ed Engl. 2017 Mar 30;56(18):5066–70. doi: 10.1002/anie.201701858 (PMC5485009; doi:10.1002/anie.201701858)
Supplement: Supplementary file 1 — Supplementary [file ANIE-56-5066-s001.pdf]

Angewandte Chemie
Eine Zeitschrift der Gesellschaft Deutscher Chemiker

Supporting Information

A Structurally Characterized Organometallic Plutonium(IV) Complex

Christos Apostolidis, Olaf Walter, Jochen Vogt,* Phil Liebing, Laurent Maron,* and Frank T. Edelmann**

anie_201701858_sm_miscellaneous_information.pdf

Supporting Information

Contents

		Page
**Synthesis and spectroscopic characterization**		**3**
**Table S1.**	^{1}H NMR and ^{13} C NMR data for **4**	4
**Figure S1.**	$^{1}H/^{13}C$-HSQC spectrum (full)	4
**Figure S2.**	Zoom into the aromatic region of the $^{1}H/^{13}C$-HSQC spectrum and the 1H-NMR spectrum	5
**Crystal structure report**		**5**
**Figure S3.**	Molecular structure of the title compound in the crystal	6
**Table S2.**	Crystallographic experimental details	7
**Table S3.**	Atomic coordinates and equivalent isotropic displacement parameters	8
**Table S4.**	Interatomic distances	9
**Table S5.**	Selected Interatomic angles	9
**Table S6.**	Anisotropic displacement parameters	10
**Table S7.**	Derived atomic coordinates and displacement parameters for hydrogen atoms	11
**Computational study details**		**12**
**Table S8**.	Structural parameters of plutonocene ($Pu(COT)_2$)	13
**Figure S4.**	Structure of plutonocene ($Pu(COT)_2$)	13
**Table S9.**	Bond lengths from *ab initio* geometry optimizations in comparison with experimental data	14
**Figure S5.**	Structure of Pu(1,3-COT'')(1,4-COT'') (**4**) from MP2/LANL2DZ(d,p) geometry optimizations	15
**Figure S6a–d**.	Molecule orbital diagrams of An(1,3-COT'')(1,4-COT'') and $An(1,4\text{-}COT'')_2$ complexes (An = U, Pu).	16
**References**		**20**

Synthesis and spectroscopic characterization

General: ***Caution!*** All Pu isotopes are radioactive. ^{239}Pu is furthermore a fissile material and underlies therefor special control, regulations and restrictions. Actinides are in general α emitters and therefore any contamination or incorporation has to be avoided for sure.

The manipulations with radionuclides were conducted in the radiochemical laboratories at the Joint Research Centre (JRC) – Karlsruhe, Germany. Unsealed transuranium compounds were manipulated in dinitrogen filled (99+%), negative-pressure radiological gloveboxes. The glovebox for preparative chemistry was fitted with an automated dual vacuum/argon manifold and standard Schlenk techniques were used. NMR spectra were recorded on the Bruker Ascent™ 400 MHz WB NMR/DNP spectrometer equipped with a Bruker Triple Resonance Broad Band Probe (TBI). Degassed fluoropolymer NMR tube liners (4 mm nominal O.D.; 140°C, 6×10^{-4} mbar, 12 h) were charged with the liquid samples ensuring that the outer surface remained free from contamination, and sealed. The sealed liner was then transferred into a standard borosilicate glass NMR tube placed in a PVC bag, which was sealed by welding. Chemical shifts were calibrated against residual protio solvent signal and are reported relative to tetramethylsilane (δ = 0 ppm).

Commercially available reagents and solvents were obtained from Sigma-Aldrich Co., ACROS Organics, STREM Chemicals Inc. or ABCR GmbH & Co. KG and used as received unless otherwise stated. Argon 6.0 N was supplied by basi Schöberl GmbH & Co. KG and purified with Agilent Technologies Big Moisture and Oxygen Traps to achieve sub-5 and sub-1 ppb levels of the corresponding impurities respectively. Anhydrous grade solvents were purified immediately before use by distillation from appropriate drying agents under argon. $PuCl_3$ was on stock but might be prepared according to (1, 2).

Compound: $[Pu\{\eta^8\text{-}C_8H_6\text{-}1,3\text{-}(SiMe_3)_2\}\{\eta^8\text{-}C_8H_6\text{-}1,4\text{-}(SiMe_3)_2\}]$ (**4**)

140 mg (0.56 mmol) $C_8H_6\text{-}1,4\text{-}(SiMe_3)_2$ were dissolved in 5 ml THF. 0.8 ml of 1.6 molar *n*-BuLi (1.28 mmol) in hexanes are added at RT. The solution turns deep orange and warms up slightly. 97.2 mg (0.28 mmol) $PuCl_3$ were added as a solid. The brown-green reaction mixture was stirred at r.t. for 7d. The solvent was removed and the residue was dissolved in 10 ml of toluene forming a green solution. Addition of 20 mg (0.15 mmol) water-free $CoCl_2$ turned the reaction dark red. It was stirred at RT for 5h and refluxed for 15h to complete the

reaction. The solvent was removed and the raw product was extracted with *n*-pentane, followed by removing the insoluble matter by filtration. Removal of the *n*-pentane from the filtrate yielded 170 mg (84%) of a waxy product which crystallised overnight under formation of dark red crystals with a low melting point of < 60°C.
Pu content: found 31.0 % (α-spectroscopy), calculated for $C_{28}H_{48}PuSi_4$: 33.1%.

Table S1. ^{1}H NMR and ^{13}C NMR data for **4** (C_6D_6, assigned via CH correlated spectroscopy).

δ ^{1}H	multiplicity	rel intensity	δ ^{13}C	Assignm.
10.19	m, br	2	94.1	CH
10.08	m, br	2	95.1	CH
9.78	m, vbr	1	95.6	CH
9.48	m, br	2	95.7	CH
0.97	m, br	18	0.6	CH_3

Figure S1. $^{1}H/^{13}C$-HSQC spectrum (full).

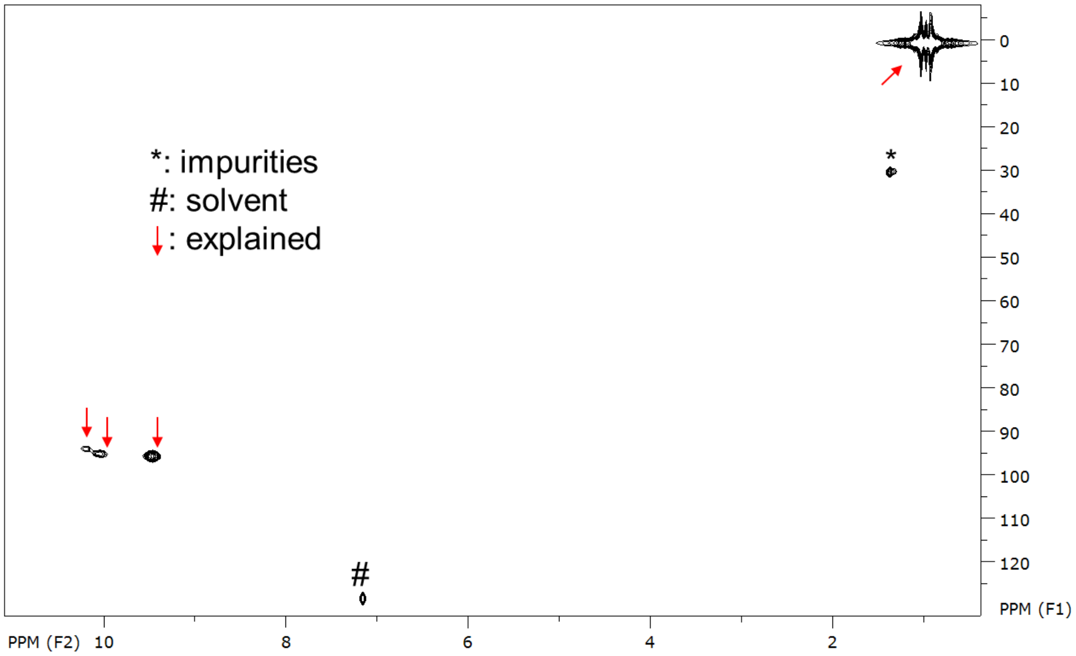

Figure S2. Zoom into the aromatic region of the $^{1}H/^{13}C$-HSQC spectrum (left) and the 1H-NMR spectrum (right). The CH correlated signal of the weak because very broad resonance at 9.78 ppm assigned to the proton in 2-position of 1,3-substituted COT ligand is resolved.

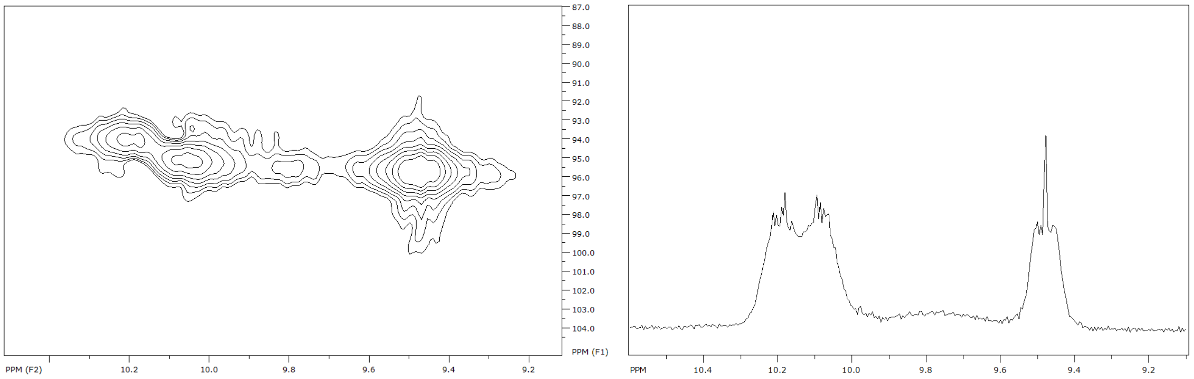

Crystal structure report

Single crystal analysis of **4** was performed on a Bruker Apex II Quazar diffractometer at 100 K collecting four spheres of data with an irradiation time of 10 to 20 s per frame applying a combination of ω– and φ–scans. For more information be referred to table S1.

The measurement was controlled with the APEX II software [11], integration of the data proceeded with SAINT [11], the data were corrected for Lorentz- and polarisation effects, and an experimental absorption correction with SADABS was performed [12]. The structures have been solved by direct methods and refined to a minimum R-value with SHELXL-2013 via full-matrix least-squares on F^2. [13]

The cif-data of the structure of **4** has been deposited at the CCDC, data are available from https://www.ccdc.cam.ac.uk/ on request [14] referring to the depository number CCDC 1049923.

Compound: $[Pu\{\eta^8\text{-}C_8H_6\text{-}1,3\text{-}(SiMe_3)_2\}\{\eta^8\text{-}C_8H_6\text{-}1,4\text{-}(SiMe_3)_2\}]$ (**4**)
Formula sum: $C_{28}H_{48}PuSi_4$

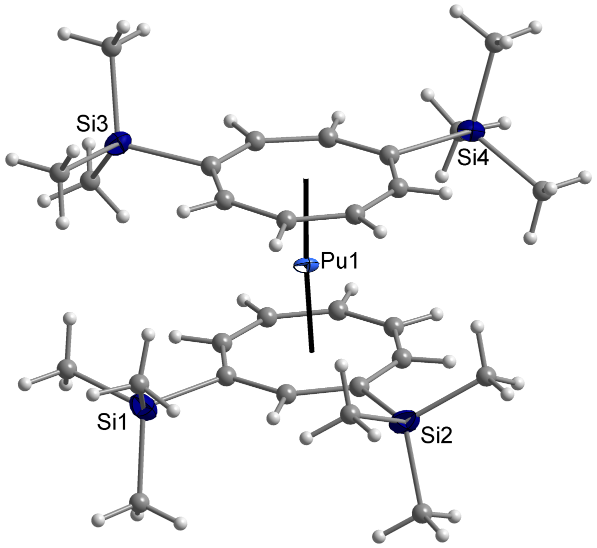

Figure S3. Molecular structure of Pu(1,3-COT'')(1,4-COT'') (**4**) in the crystal. Displacement ellipsoids of Pu and Si drawn at the 50% probability level, C and H atoms as spheres of arbitrary size.

Table S2. Crystallographic experimental details.

A. Crystal data	
formula	$C_{28}H_{48}PuSi_4$
formula weight	739.02
crystal dimensions (mm)	0.067 x 0.057 x 0.009
crystal system	monoclinic
space group	$P2_1/c$
unit cell parameters[a]	
a (Å)	22.385(5)
b (Å)	11.481(3)
c (Å)	12.753(3)
β (deg)	102.208(3)
V (Å^3)	3204(1)
Z	4
ρ_{calcd} (g cm^{-3})	1.532
μ (mm^{-1})	2.222
B. Data collection and structure refinement	
diffractometer	Bruker Apex II Quazar
radiation (λ [Å])	graphite-monochromated Mo-K_α (0.71073)
temperature (°C)	–170
scan type	ω and φ scans
completeness of dataset	100%
data collection θ limit (deg)	24.999
total data collected	19701 ($-26 \le h \le 26$, $-13 \le k \le 8$, $-15 \le l \le 15$)
independent reflections	5641 (R_{int} = 0.1476)
independent reflections with $I>2\sigma(I)$	3296
structure solution	heavy atom methods (SHELXS 2013)
refinement method	full-matrix least-squares on F^2 (SHELXL 2016/4)
absorption correction method	multi-scan
range of transmission factors	0.8654–0.9803
data/restraints/parameters	5641 / 108 / 316
goodness-of-fit (S) [all data]	0.983
final R indices	
R_1 [all data, $I \ge 2\sigma(I)$]	0.1373, 0.0640
wR_2 [all data, $I \ge 2\sigma(I)$]]	0.1301, 0.1^072
largest difference peak and hole	2.516 and –1.883 e Å^{-3}
Extinction coefficient	–
Flack parameter	–

Refinement special details: Restraints on the anisotropic displacement parameters of the COT ring C atoms (C1–C16) have been applied (SIMU and ISOR commands in SHELXL, with an ESD of each 0.01).

Table S3. Atomic Coordinates and Equivalent Isotropic Displacement Parameters.

Atom	*x*	*y*	*z*	U_{eq}, Å^2
PU1	0.26867(2)	0.09089(4)	0.45595(4)	0.01798(15)
SI1	0.10488(16)	–0.0960(4)	0.3352(3)	0.0323(9)
SI2	0.31341(18)	–0.2455(4)	0.5734(3)	0.0332(10)
SI3	0.12051(17)	0.3419(3)	0.4290(3)	0.0257(9)
SI4	0.43532(16)	0.2711(3)	0.5596(3)	0.0257(9)
C1	0.1872(5)	–0.0547(11)	0.3442(9)	0.022(3)
C2	0.2295(6)	–0.1235(11)	0.4217(10)	0.025(3)
C3	0.2931(6)	–0.1343(11)	0.4615(10)	0.024(3)
C4	0.3433(5)	–0.0784(12)	0.4305(10)	0.026(3)
C5	0.3525(6)	0.0075(11)	0.3563(10)	0.026(3)
C6	0.3140(5)	0.0729(11)	0.2798(9)	0.021(3)
C7	0.2510(5)	0.0830(12)	0.2447(9)	0.024(3)
C8	0.1999(6)	0.0330(11)	0.2716(10)	0.024(3)
C9	0.1945(5)	0.2662(12)	0.4922(9)	0.023(3)
C10	0.2453(6)	0.3159(12)	0.4621(10)	0.028(3)
C11	0.3098(6)	0.3032(11)	0.4896(10)	0.027(3)
C12	0.3538(6)	0.2366(12)	0.5618(10)	0.026(3)
C13	0.3455(6)	0.1491(12)	0.6360(10)	0.026(3)
C14	0.2952(5)	0.0955(13)	0.6684(9)	0.028(3)
C15	0.2344(6)	0.1045(13)	0.6383(10)	0.032(3)
C16	0.1899(5)	0.1749(12)	0.5670(10)	0.025(3)
C17	0.0520(6)	0.0114(12)	0.2548(12)	0.039(4)
C18	0.0865(6)	–0.1060(16)	0.4711(11)	0.056(5)
C19	0.0916(6)	–0.2405(12)	0.2669(12)	0.048(4)
C20	0.3861(6)	–0.2094(14)	0.6662(11)	0.053(5)
C21	0.3209(7)	–0.3858(12)	0.5068(12)	0.048(4)
C22	0.2531(6)	–0.2552(14)	0.6538(11)	0.048(4)
C23	0.1276(6)	0.4990(12)	0.4718(11)	0.038(4)
C24	0.0538(5)	0.2800(14)	0.4737(10)	0.038(4)
C25	0.1075(6)	0.3359(12)	0.2793(10)	0.030(4)
C26	0.4433(6)	0.3226(15)	0.4244(11)	0.046(5)
C27	0.4850(6)	0.1398(12)	0.5977(12)	0.044(4)
C28	0.4618(6)	0.3886(11)	0.6576(10)	0.030(4)

Table S4. Interatomic Distances (Å).

atom1	atom2	distance		atom1	atom2	distance
PU1	C1	2.656(11)		SI3	C24	1.849(13)
PU1	C2	2.618(12)		SI3	C25	1.871(12)
PU1	C3	2.640(13)		SI4	C12	1.873(13)
PU1	C4	2.628(13)		SI4	C26	1.868(14)
PU1	C5	2.658(13)		SI4	C27	1.874(14)
PU1	C7	2.640(11)		SI4	C28	1.850(13)
PU1	C8	2.609(12)		C1	C2	1.450(16)
PU1	C10	2.641(14)		C1	C8	1.437(17)
PU1	C11	2.608(13)		C2	C3	1.410(16)
PU1	C13	2.647(11)		C3	C4	1.422(17)
PU1	C14	2.648(12)		C4	C5	1.412(17)
PU1	C15	2.604(13)		C5	C6	1.380(16)
SI1	C1	1.883(12)		C6	C7	1.392(15)
SI1	C17	1.860(14)		C7	C8	1.384(16)
SI1	C18	1.867(14)		C9	C10	1.396(17)
SI1	C19	1.869(15)		C9	C16	1.436(17)
SI2	C3	1.897(13)		C10	C11	1.420(16)
SI2	C20	1.846(13)		C11	C12	1.422(17)
SI2	C21	1.845(14)		C12	C13	1.419(18)
SI2	C22	1.864(14)		C13	C14	1.418(17)
SI3	C9	1.893(12)		C14	C15	1.338(15)
SI3	C23	1.881(13)		C15	C16	1.446(17)

Table S5. Selected Interatomic Angles (deg.).

atom1	atom2	atom3	angle		atom1	atom2	atom3	angle
C1	SI1	C17	111.5(6)		C24	SI3	C25	109.8(6)
C1	SI1	C18	111.3(6)		C12	SI4	C26	111.4(6)
C1	SI1	C19	107.9(6)		C12	SI4	C27	110.6(6)
C17	SI1	C18	108.3(7)		C12	SI4	C28	108.6(6)
C17	SI1	C19	108.2(7)		C26	SI4	C27	109.1(7)
C18	SI1	C19	109.5(8)		C26	SI4	C28	108.2(7)
C3	SI2	C20	111.7(6)		C27	SI4	C28	108.9(6)
C3	SI2	C21	105.8(6)		SI1	C1	C2	113.2(9)
C3	SI2	C22	111.6(6)		SI1	C1	C8	117.5(9)
C20	SI2	C21	109.7(7)		C2	C1	C8	129.1(11)
C20	SI2	C22	107.7(7)		C1	C2	C3	139.2(12)
C21	SI2	C22	110.3(7)		SI2	C3	C2	113.2(10)
C9	SI3	C23	107.7(6)		SI2	C3	C4	115.7(9)
C9	SI3	C24	112.5(6)		C2	C3	C4	131.1(12)
C9	SI3	C25	110.5(6)		C3	C4	C5	137.5(12)
C23	SI3	C24	107.7(7)		C4	C5	C6	134.1(12)
C23	SI3	C25	108.5(6)		C5	C6	C7	135.5(12)

Table S5 (continued). Selected Interatomic Angles (deg.).

atom1	atom2	atom3	angle		atom1	atom2	atom3	angle
C6	C7	C8	136.0(13)		SI4	C12	C11	114.9(10)
C1	C8	C7	137.4(12)		SI4	C12	C13	115.2(9)
SI3	C9	C10	112.6(10)		C11	C12	C13	129.9(12)
SI3	C9	C16	116.0(9)		C12	C13	C14	136.5(12)
C10	C9	C16	131.2(12)		C13	C14	C15	134.8(14)
C9	C10	C11	137.3(14)		C14	C15	C16	138.4(14)
C10	C11	C12	138.2(13)		C9	C16	C15	133.6(12)

Table S6. Anisotropic Displacement Parameters (U_{ij}, Å^2)

atom	U_{11}	U_{22}	U_{33}	U_{23}	U_{13}	U_{12}
PU1	0.0279(2)	0.0086(2)	0.0172(2)	–0.0008(3)	0.00409(16)	–0.0005(3)
SI1	0.030(2)	0.030(2)	0.035(2)	0.001(3)	0.0027(16)	–0.009(2)
SI2	0.044(2)	0.014(2)	0.039(3)	0.008(2)	0.0013(19)	0.0017(19)
SI3	0.032(2)	0.020(2)	0.026(2)	0.003(2)	0.0074(17)	0.0042(18)
SI4	0.032(2)	0.019(2)	0.026(2)	–0.003(2)	0.0058(16)	–0.0029(18)
C1	0.032(6)	0.012(6)	0.021(5)	–0.004(5)	0.002(4)	–0.005(5)
C2	0.038(6)	0.010(6)	0.024(5)	–0.005(5)	–0.001(5)	–0.012(5)
C3	0.038(5)	0.006(5)	0.024(5)	–0.005(5)	–0.001(4)	–0.001(4)
C4	0.033(5)	0.010(6)	0.032(6)	–0.002(5)	0.001(5)	0.005(5)
C5	0.033(6)	0.015(6)	0.033(6)	–0.003(5)	0.013(5)	–0.003(5)
C6	0.035(5)	0.007(6)	0.026(5)	–0.006(5)	0.016(4)	–0.006(5)
C7	0.040(6)	0.014(6)	0.020(5)	–0.005(6)	0.008(4)	–0.006(5)
C8	0.035(6)	0.013(6)	0.024(5)	–0.004(5)	0.009(5)	–0.004(5)
C9	0.024(5)	0.023(6)	0.021(5)	–0.006(5)	0.006(5)	–0.002(5)
C10	0.036(6)	0.021(6)	0.026(6)	–0.008(5)	0.006(5)	0.005(5)
C11	0.041(6)	0.012(6)	0.029(6)	–0.011(5)	0.007(5)	–0.005(5)
C12	0.037(6)	0.013(6)	0.025(6)	–0.014(5)	0.001(5)	0.002(5)
C13	0.032(6)	0.018(6)	0.022(6)	–0.011(5)	–0.007(5)	0.002(5)
C14	0.029(5)	0.031(6)	0.020(5)	–0.009(6)	–0.001(4)	–0.001(6)
C15	0.028(5)	0.034(7)	0.030(6)	–0.011(6)	–0.003(5)	–0.011(6)
C16	0.024(5)	0.025(6)	0.026(6)	–0.009(5)	0.007(5)	–0.011(5)
C17	0.031(8)	0.029(10)	0.059(10)	–0.003(9)	0.009(7)	–0.004(7)
C18	0.046(9)	0.072(14)	0.053(10)	0.003(11)	0.019(7)	–0.010(10)
C19	0.046(10)	0.022(10)	0.077(12)	0.009(10)	0.011(8)	–0.007(8)
C20	0.049(10)	0.052(12)	0.049(10)	0.024(10)	–0.014(8)	0.007(9)
C21	0.077(10)	0.013(9)	0.055(9)	0.005(8)	0.013(8)	–0.003(7)
C22	0.067(11)	0.029(10)	0.043(10)	0.026(9)	0.004(8)	–0.005(8)
C23	0.055(10)	0.024(9)	0.028(8)	–0.016(8)	–0.004(7)	0.014(7)
C24	0.028(8)	0.055(11)	0.030(8)	–0.001(8)	0.008(6)	–0.001(8)
C25	0.037(8)	0.024(9)	0.030(8)	0.011(8)	0.008(6)	0.004(7)
C26	0.021(8)	0.067(13)	0.050(10)	–0.001(10)	0.008(7)	–0.001(8)
C27	0.038(9)	0.033(10)	0.058(11)	–0.016(9)	0.003(7)	0.006(7)
C28	0.034(8)	0.012(9)	0.042(8)	0.002(7)	0.000(6)	0.006(6)

Table S7. Derived Atomic Coordinates and Displacement Parameters for Hydrogen Atoms.

Atom	*x*	*y*	*z*	U_{eq}, Å^2
H2	0.207959	-0.177622	0.456126	0.030
H4	0.381147	-0.106662	0.470906	0.031
H5	0.394503	0.023973	0.359843	0.031
H6	0.335713	0.123174	0.241960	0.026
H7	0.240271	0.137863	0.188082	0.029
H8	0.163304	0.065746	0.230954	0.029
H10	0.233438	0.374139	0.408791	0.034
H11	0.329035	0.353506	0.447676	0.033
H13	0.383360	0.118441	0.673767	0.031
H14	0.307568	0.040368	0.724330	0.033
H15	0.214773	0.048438	0.674311	0.038
H16	0.148881	0.156954	0.570307	0.030
H17A	0.058298	0.012486	0.181081	0.047
H17B	0.060175	0.089039	0.286595	0.047
H17C	0.009658	-0.010653	0.254404	0.047
H18A	0.042460	-0.117400	0.463744	0.067
H18B	0.099022	-0.033934	0.510889	0.067
H18C	0.108528	-0.172056	0.510132	0.067
H19A	0.049779	-0.266375	0.265623	0.058
H19B	0.120606	-0.297518	0.305902	0.058
H19C	0.097594	-0.233392	0.193171	0.058
H20A	0.393986	-0.265954	0.725101	0.064
H20B	0.383505	-0.131030	0.695353	0.064
H20C	0.419553	-0.212068	0.627496	0.064
H21A	0.355432	-0.381963	0.470851	0.058
H21B	0.283242	-0.401725	0.453634	0.058
H21C	0.327866	-0.448181	0.560402	0.058
H22A	0.264261	-0.315234	0.709094	0.057
H22B	0.213977	-0.275670	0.606549	0.057
H22C	0.249218	-0.179933	0.687915	0.057
H23A	0.091140	0.541733	0.435728	0.045
H23B	0.163889	0.533078	0.452293	0.045
H23C	0.131495	0.503892	0.549668	0.045
H24A	0.016774	0.322218	0.439197	0.045
H24B	0.059777	0.287503	0.551737	0.045
H24C	0.049460	0.197551	0.453696	0.045
H25A	0.133695	0.275653	0.258235	0.036
H25B	0.117543	0.411598	0.251925	0.036
H25C	0.064589	0.317342	0.249387	0.036
H26A	0.422893	0.398132	0.409020	0.055
H26B	0.424468	0.265946	0.369888	0.055
H26C	0.486733	0.330922	0.423233	0.055
H27A	0.527419	0.159625	0.596671	0.053
H27B	0.471102	0.076852	0.546466	0.053

Table S7 (continued). Derived Atomic Coordinates and Displacement Parameters for Hydrogen Atoms.

Atom	*x*	*y*	*z*	U_{eq}, Å^2
H27C	0.482338	0.114336	0.669954	0.053
H28A	0.434741	0.456157	0.640695	0.037
H28B	0.503588	0.411098	0.654001	0.037
H28C	0.461234	0.360895	0.730064	0.037

Computational study details

In view of the low point symmetry and the considerable dimension of the Pu(1,3-COT'')(1,4-COT'') molecule, compromises in the selection of basis sets and the chosen methods had to be made. Thus, second order Møller-Plesset perturbation theory (MP2), covering significant parts of dynamic electron correlation, in combination with restricted open shell Hartree Fock (ROHF) single reference calculations were considered most appropriate for geometry optimizations of Pu(1,3-COT'')(1,4-COT'') (**4**). MP2 energies and gradients were obtained with Z-averaged perturbation theory (ZAPT)[3,4] available in the GAMESS (US) code.[5] In addition, effective core potentials (ECP) were used for the elements plutonium and silicon in combination with LANL2DZ basis sets (Los Alamos National Laboratory 2 double ζ) for all elements.[6-8] The effective core potentials replace 118 of 366 electrons. At the same time relativistic effects predominantly affecting the core shells of plutonium are considered in a scalar relativistic approximation within the ECPs. As has been found for plutonocene,[9] spin-orbit coupling is not expected to have large influence on the equilibrium geometry of Pu(1,3-COT'')(1,4-COT''). The carbon basis set was augmented with additional polarization and diffuse functions, denoted LANL2DZ(d,p) in the following. On the MP2 level this modification significantly improved the plutonium carbon bond length in geometry optimizations of the symmetric plutonocene molecule, as can be seen in Table S7. For Pu(1,3-COT'')(1,4-COT'') (**4**), the ground state was assumed to have the same multiplicity as plutonocene with ground state ^5A_g.[9,10]

Table S8: Structural parameters of plutonocene ($Pu(COT)_2$) derived from quantum chemical calculations using a modified LANL2DZ basis and effective core potentials, in comparison with high level *ab initio* results from refs. (7) and (8). The dihedral angle CCH smaller than 180° indicates a bending of the C-H bond towards the Pu atom (see Fig. 1).

Method	Pu-COT (pm)	C-C (Å)	C-H (Å)	CCH (deg.)
ROHF/LANL2DZ/ECP	201.9	1.416	1.072	176.1
ROHF/ LANL2DZ(d,p)/ECP	194.5	1.409	1.075	174.4
MP2 LANL2DZ(d,p)/ECP	188.7	1.423	1.091	173.2
CASPT2 [7,8]	189.8	1.404	1.087	180.0

Figure S4: Structure of plutonocene ($Pu(COT)_2$), optimized in quantum chemical calculations on the MP2/LANL2DZ(d,p) level.

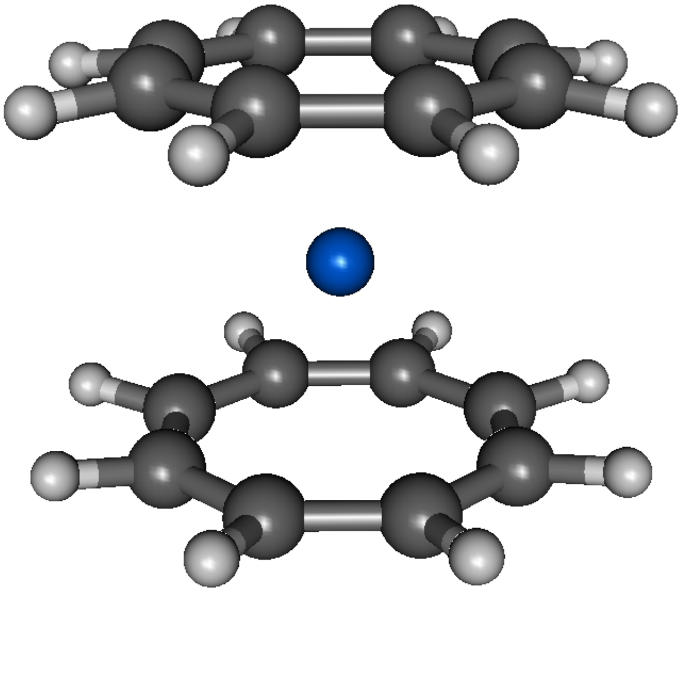

Table S9: Bond lengths (Å) from *ab initio* geometry optimizations of the Pu(1,3-COT'')(1,4-COT'') molecule in comparison with experimental data. For the labeling of the atoms see Fig. S3. Ring(1,3) is the COT'' ring with the $SiMe_3$ groups positioned at carbons C(1) and C(3).

Bond	ROHF/LANL2DZ(d,p) / ECP	MP2/LANL2DZ(d,p) / ECP	Crystal Structure
Ring centroids			
Ring(9,12)-Pu	1.981	1.842	1.904
Ring(1,3)-Pu	1.978	1.842	1.889
Centroid-Pu-Centroid angle (°)	1.779	1.772	1.766
Ring(1,3)			
C(1)-C(2)	1.425	1.431	1.450(16)
C(2)-C(3)	1.411	1.428	1.410(16)
C(3)-C(4)	1.423	1.427	1.422(17)
C(4)-C(5)	1.407	1.413	1.412(17)
C(5)-C(6)	1.400	1.418	1.380(16)
C(6)-C(7)	1.412	1.420	1.392(15)
C(7)-C(8)	1.402	1.421	1.384(16)
C(8)-C(1)	1.417	1.426	1.437(17)
Ring (9,12)			
C(9)-C(10)	1.425	1.429	1.396(17)
C(10)-C(11)	1.405	1.422	1.420(16)
C(11)-C(12)	1.417	1.427	1.422(17)
C(12)-C(13)	1.422	1.428	1.419(18)
C(13)-C(14)	1.399	1.421	1.418(17)
C(14)-C(15)	1.413	1.419	1.338(15)
C(15)-C(16)	1.407	1.422	1.446(17)
C(16)-C(9)	1.409	1.426	1.436(17)
Ring-Si			
Si(1)-C(1)	1.915	1.906	1.883(12)
Si(2)-C(3)	1.917	1.905	1.897(13)
Si(3)-C(9)	1.913	1.908	1.893(12)
Si(4)-C(12)	1.912	1.907	1.873(13)
Si-Methyl			
Si(1)-C(17)	1.899	1.895	1.860(14)
Si(1)-C(18)	1.896	1.893	1.867(14)
Si(1)-C(19)	1.898	1.897	1.869(15)
Si(2)-C(20)	1.898	1.894	1.846(13)
Si(2)-C(21)	1.896	1.897	1.845(14)
Si(2)-C(22)	1.897	1.896	1.864(14)

Table S9 (continued): Bond lengths (Å) from *ab initio* geometry optimizations of the Pu(1,3-COT'')(1,4-COT'') molecule in comparison with experimental data. For the labeling of the atoms see Fig. S3.

Bond	ROHF/LANL2DZ(d,p) / ECP	MP2/LANL2DZ(d,p) / ECP	Crystal Structure
Si(3)-C(23)	1.897	1.897	1.881(13)
Si(3)-C(24)	1.896	1.893	1.849(13)
Si(3)-C(25)	1.897	1.896	1.871(12)
Si(4)-C(26)	1.898	1.896	1.868(14)
Si(4)-C(27)	1.898	1.895	1.874(14)
Si(4)-C(28)	1.897	1.899	1.850(13)

Figure S5. Structure of Pu(1,3-COT'')(1,4-COT'') (**4**) from MP2/LANL2DZ(d,p) geometry optimizations. The black dots linked by red dashed lines with the central Pu atom mark the positions of the ring centroids.

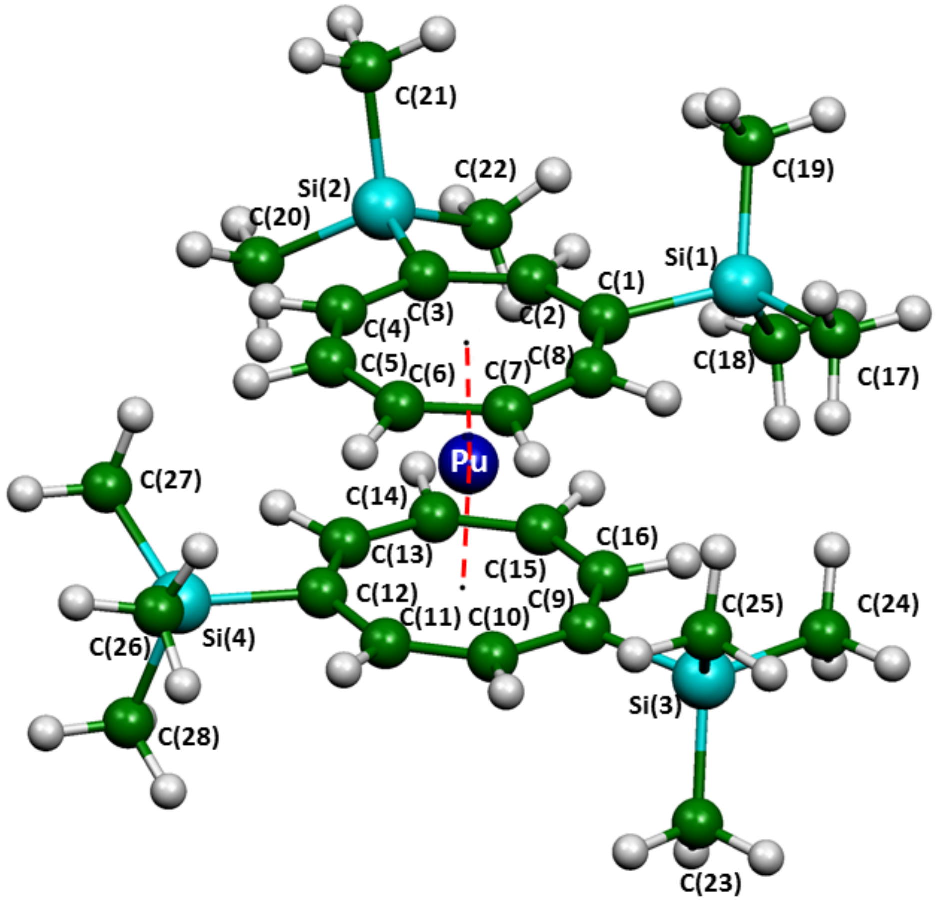

Figure S6a–d. Molecule orbital diagrams of An(1,3-COT'')(1,4-COT'') and An(1,4-COT'')$_2$ complexes (An = U, Pu).

a) The geometry equivalent to the crystal structure of Pu(1,3-COT'')(1,4-COT'') (**4**).

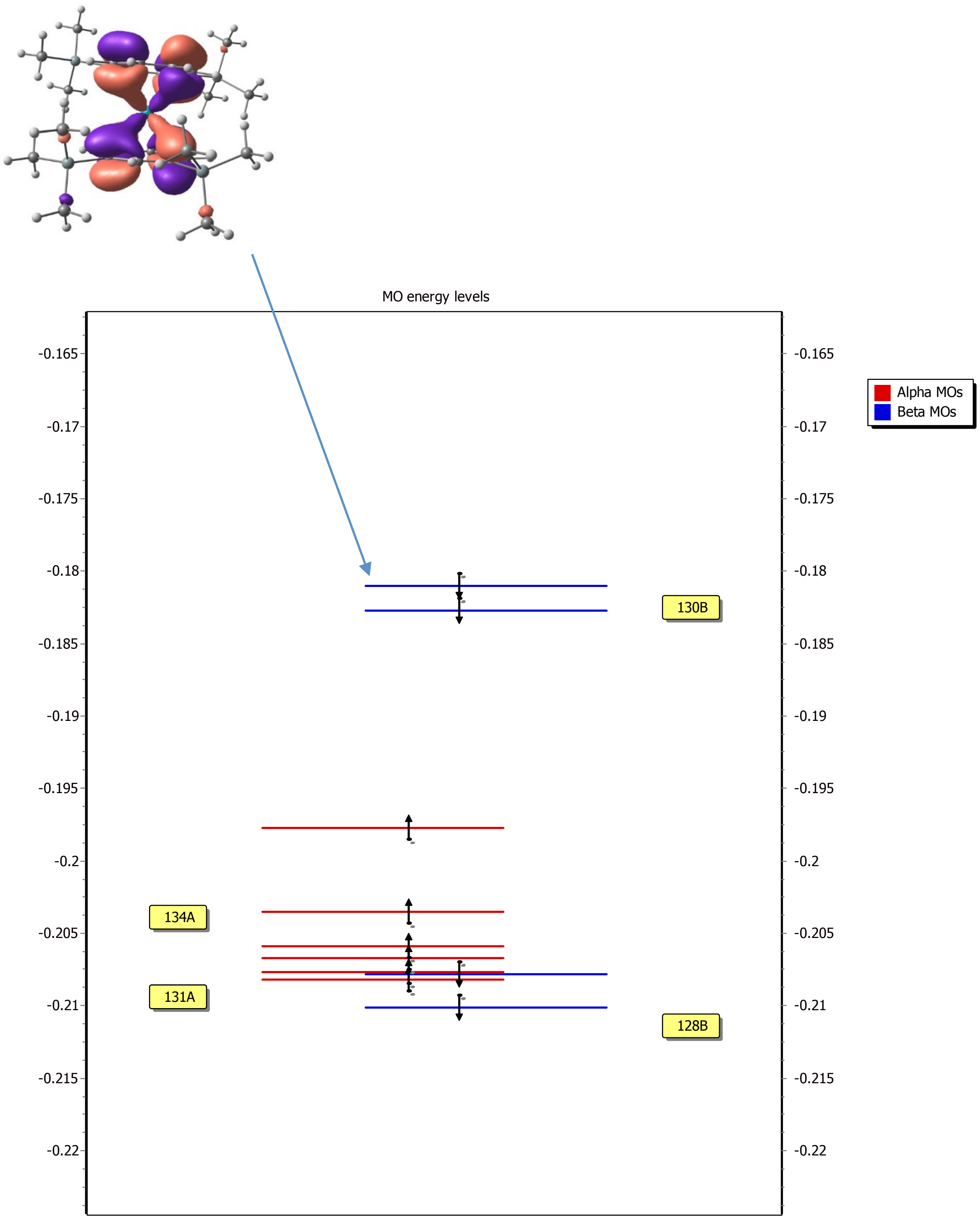

b) The geometry equivalent of hypothetical Pu(1,4-COT'')$_2$ to the uranocene analogue from (15).

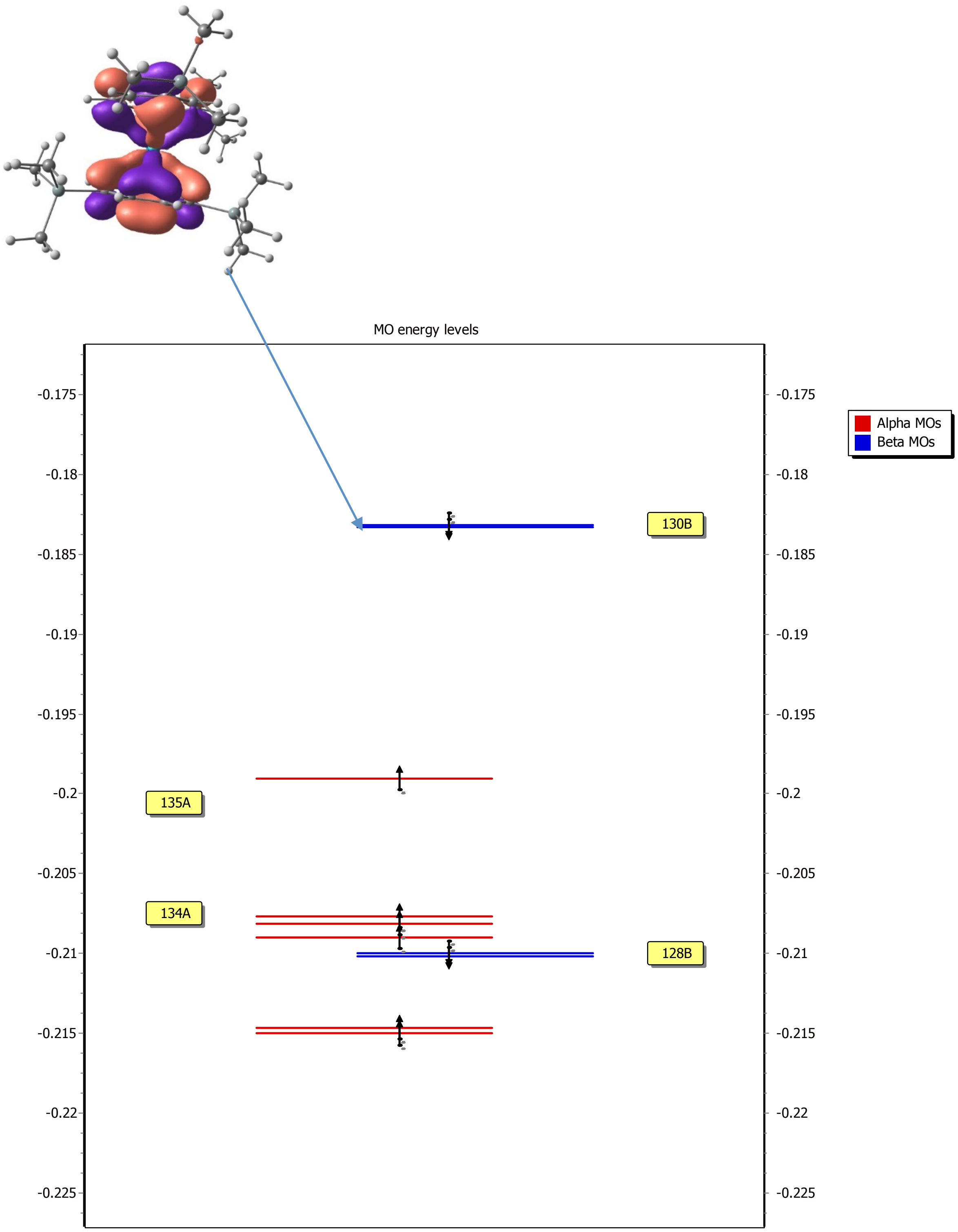

c) The geometry equivalent of hypothetical U(1,3-COT'')(1,4-COT'') to the plutonocene analogue **4**.

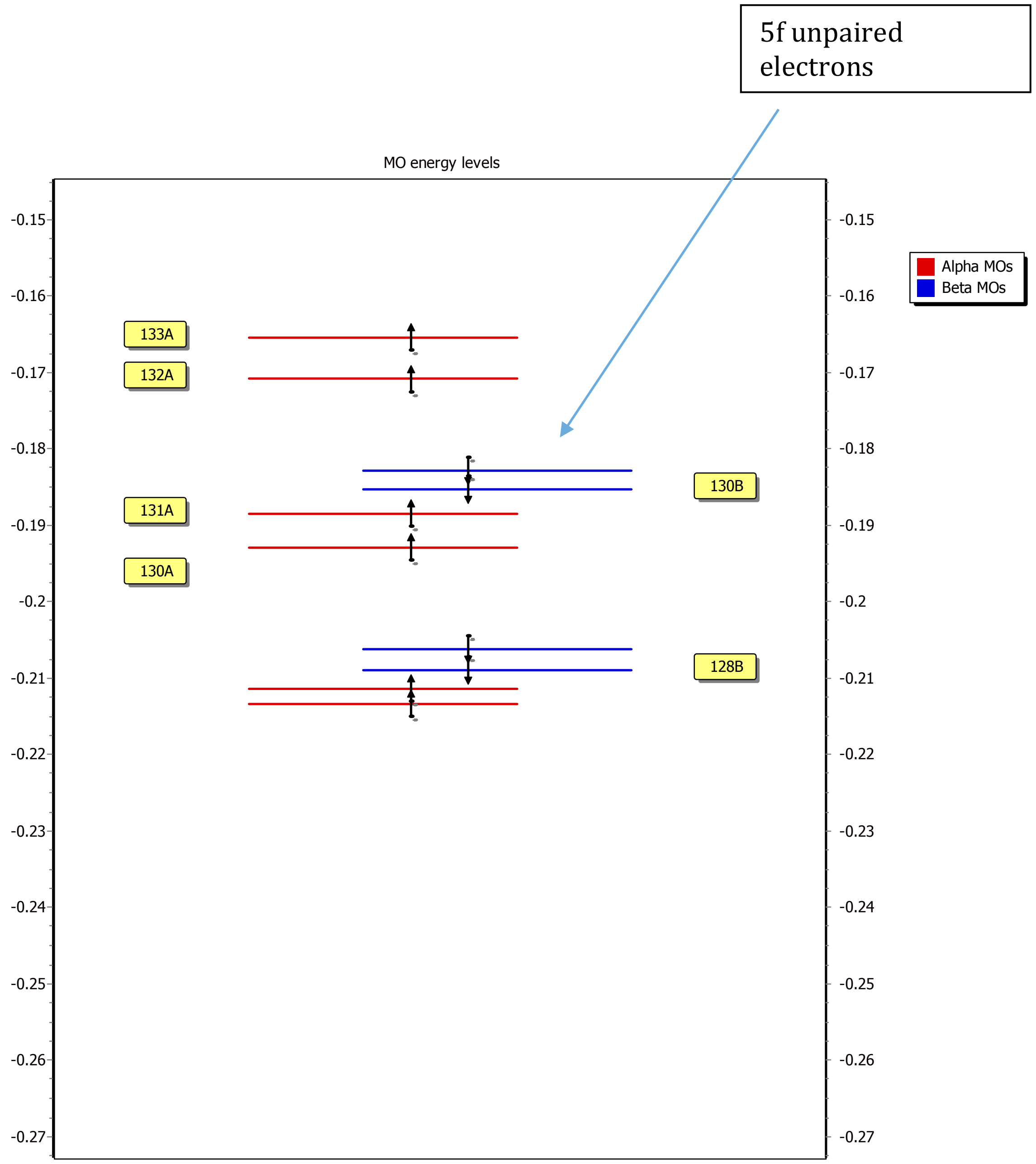

d) The geometry equivalent to the crystal structure of U(1,4-COT'')$_2$ from (15).

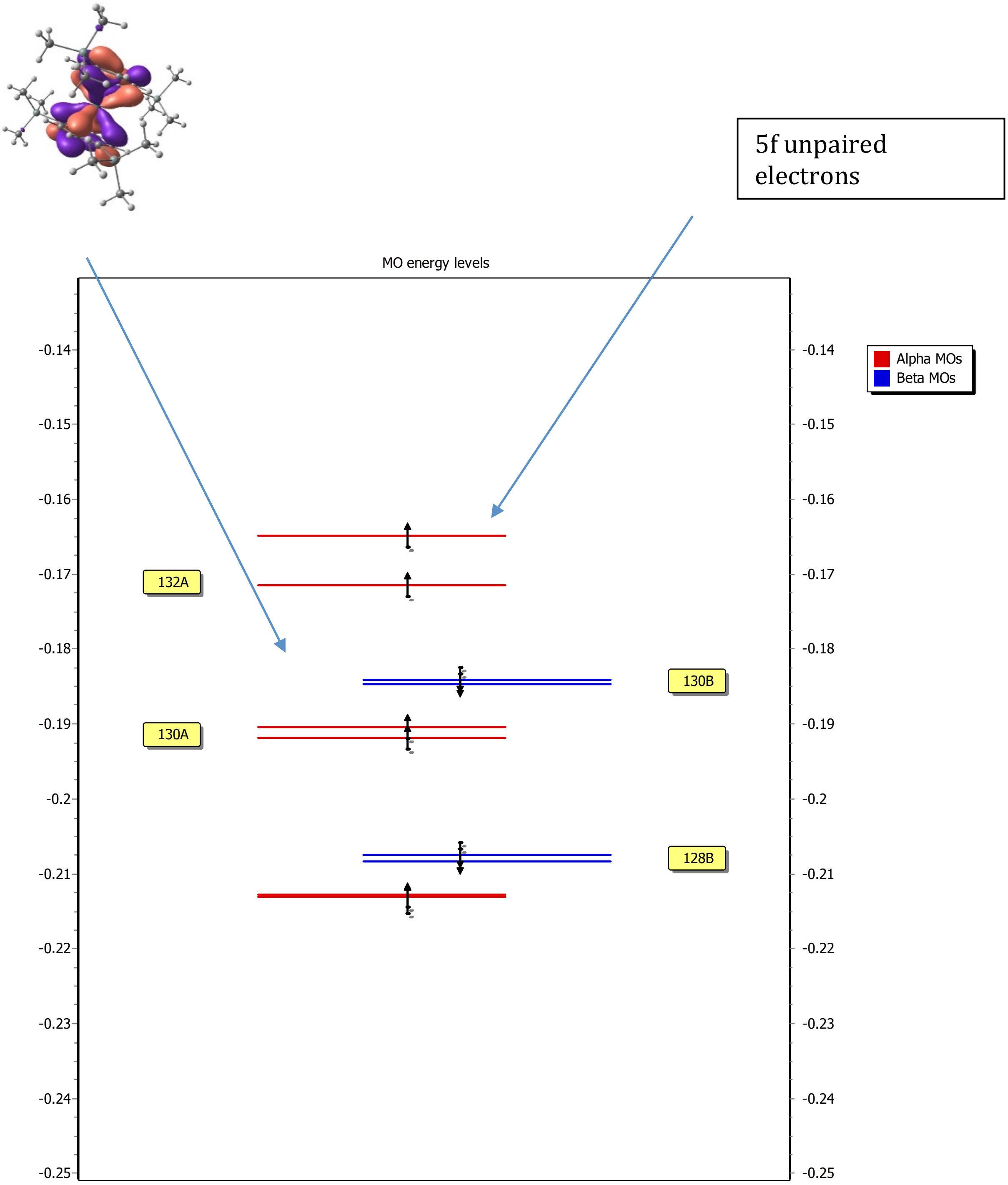

References

(1) F. Baumgärtner, E. O. Fischer, B. Kanellakopulos, P. Laubereau, *Angew. Chem.* **1965**, *77*, 866-867.

(2) David L. Clark, Siegfried S. Hecker, Gordon D. Jarvinen, and Mary P. Neu in *The Chemistry of the Actinide and Transactinide Elements*, 4th edition, (Eds: L. R. Morss, N. M. Edelstein, J. Fuger), Springer Netherlands, **2010**, 1092.

(3) Lee, T. J.; Jayatilaka, D. *Chem. Phys. Lett.* **1993**, *201*, 1.

(4) Lee, T. J.; Rendell, A. P.; Dyall, K. G.; Jayatilaka, D. *J. Chem. Phys*. **1994**, *100*, 7400.

(5) Schmidt, M. W., Baldridge, K. K.; Boatz, J. A.; Elbert, S. T.; Gordon, M. S.; Jensen, J. H.; Koseki, S.; Matsunaga, N.; Nguyen, K. A.; Su, S. J.; Windus, T. L.; Dupuis, M.; Montgomery, J. A. *J. Comput. Chem.* **1993**, *14*, 1347.

(6) Hay, P. J.; Wadt, W. R. *J. Chem. Phys.* **1985**, *82*, 270.

(7) Hay, P. J.; Martin, R. L. *J. Chem. Phys.* **1998**, *109*, 3875.

(8) The basis sets and ECPs were taken from EMSL basis set exchange library (https://bse.pnl.gov/bse/portal).

(9) Kerridge, A.; Kaltsoyannis, N. *J. Phys. Chem. A* **2009**, *113*, 8737.

(10) Kerridge, A. *Dalton Trans.* **2013**, *42*, 16428.

(11) Bruker. APEXII, SAINT, Bruker AXS Inc., Madison, Wisconsin, USA, 2012.

(12) G. M. Sheldrick, SADABS, University of Göttingen, Germany, 1996.

(13) G. M. Sheldrick, Acta Cryst., 2015, C71, 3-8.

(14) The Cambridge Structural Database (CSD). https://www.ccdc.cam.ac.uk/

(15) Le Roy, J. J.; Gorelsky, S. I.; Korobkov, I.; Murugesu, M. *Organometallics* **2015**, *34*, 1415–1418.
